# Spine deformities in patients with Ehlers-Danlos syndrome, type IV - late results of surgical treatment

**DOI:** 10.1186/1748-7161-5-26

**Published:** 2010-11-25

**Authors:** Barbara Jasiewicz, Tomasz Potaczek, Maciej Tesiorowski, Krzysztof Lokas

**Affiliations:** 1 Jagiellonian University, College of Medicine, Department of Orthopaedics and Rehabilitation, 34-500 Zakopane, Balzera Street 15, Poland

## Abstract

**Background:**

Spinal deformities in Ehlers-Danlos syndrome are usually progressive and may require operative treatment. There is limited number of studies describing late results of surgery in this disease.

**Methods:**

This is a retrospective study of the records of 11 patients with Ehlers-Danlos syndrome type IV, treated surgically between 1990 and 2007. All patients underwent surgical treatment for spinal deformity. Duration of operation, type of instrumentation, intraoperative blood loss, complications and number of additional surgeries were noted. Radiographic measurement was performed on standing AP and lateral radiographs acquired before surgery, just after and at final follow up.

**Results:**

The mean follow up period was 5.5 ± 2.9 years (range 1-10 years). The mean preoperative thoracic and lumbar curve were 109.5 ± 19.9° (range 83° - 142°) and 75.6 ± 26.7° (range 40° - 108°) respectively. Posterior spine fusion alone was performed on 6 patients and combined anterior and posterior fusion (one- or two stage) on 5 cases. Posterior segmental spinal instrumentation was applied with use of hooks, screws and wires. The mean postoperative thoracic and lumbar curve improved to 79.3 ± 16.1° (range 56° - 105°) and 58.5 ± 27.7° (range 10° - 95°) respectively, with a slight loss of correction during follow up. The average thoracic and lumbar correction was 26.4 ± 14.9% (range 5.3 - 50.4%) and 26.3 ± 21.2% (range 7.9 - 75%). Postoperatively, the mean kyphosis was 79.5 ± 40.3° (range 21° -170°), and lordosis was 50.8 ± 18.6° (range 20° -79°). Hyperkyphosis increased during follow up while lordosis remained stable. Mean Th12-L2 angle was -3.5 ±9.9° (range -19° - 15°) postoperatively and did not change significantly during follow up.

**Conclusions:**

Huge spinal deformities in patients with Ehlers-Danlos syndrome require complex and extensive surgery. There is a big risk of sagittal imbalance in this group.

## Background

Ehlers-Danlos syndrome is a group of different inherited diseases caused by various defects of collagen metabolism [1]. It is characterized by joint hypermobility and joint laxity. From the description of the first patient by van Meckeren in 1682, it had to pass over 200 years until Ehlers and Danlos published their works and disease called from their names was identified. Current classification consists of 6 types with different genetic abnormalities and various clinical manifestations [2]. Kyphoscoliotic type (type IV) with the deficiency of lysyl hydroxylase, a collagen modifying enzyme, is of the most interest to orthopedists. Patients suffer from generalized joint laxity, muscle hypotonia at birth, progressive scoliosis and fragility of sclera with risk of ocular globe rupture. Spinal deformity, which typically involves both thoracic and lumbar spine, occurs as scoliosis or kyphoscoliosis and is visible just after birth [3]. Curve progression is rapid and operative treatment is often indicated, however, surgery is not such a simple procedure as in idiopathic scoliosis [4,5].

Surgery is much more difficult due to stiff deformity and is connected with significant risk of serious complications [5,6]. That is why individual approach to the treatment of these patients may be advisable. However, there are limited number of studies describing late results of operative treatment in patients with Ehlers Danlos syndrome.

The objective of this study was retrospective analysis of spinal deformities in patients with Ehlers Danlos syndrome. In addition we evaluated late results of operative treatment.

## Methods

This is a retrospective study of the records of patients with Ehlers Danlos syndrome type IV, treated surgically between 1990 and 2007. Material consists of 11 patients: 5 females and 6 males. The mean age at the onset of deformity was 1.6 ± 0.9 years (range 1-2.5years).

All patients had plano-valgus feet, treated operatively in 2 cases. Pectus excavatum was observed in 6 cases, pectus carinatum - in 1 case. Two patients underwent surgical correction of chest deformity. Scoliosis was present in all cases, in all but two it was kyphoscoliosis. Lenke classification was used to categorize the curve types, so type 1 was present in 7 cases, type 3 - in 3 cases and type 5 - in 1 case.

All patients underwent surgical treatment for spinal deformity.

We reviewed preoperative, intraoperative and postoperative records of all patients and noted duration of operation, type of instrumentation, intraoperative blood loss, complications and number of additional surgeries.

Radiographic measurement was performed on standing AP and lateral radiographs acquired before surgery, just after and at final follow up. Preoperative coronal curve flexibility was assessed with use of elongation radiographs. We analyzed: structural curve magnitude according to Cobb, apical vertebral translation (AVT), distance between Th1 and central sacral line (Th1-CSL), trunk shift, thoracic kyphosis and lumbar lordosis, and Th12-L2 angle.

Due to a small number of patients only basic statistical analysis was performed, data are presented as proportions (%) or as mean with standard deviations and range.

## Results

All patients underwent last clinical and radiographic examination after puberty. The mean follow up period was 5.5 ± 2.9 years (range 1-10 years) and mean age at latest examination was 19.3 ± 3.9 years (range 14.2-24.8 years).

### Before spine fusion

The mean age at the first radiogram (available for authors) was 7 ± 5 years (range 2-15 years). The mean Cobb angle of the thoracic curve was 76.3 ± 37.5° (range 11° -142°) and mean angle of the lumbar curve was 60.9 ± 24,2° (range 38° -108°). All patients underwent various non-effective conservative treatment: different exercises and braces (outside our hospital). Spine correction without fusion according to Moe was applied in 5 cases. These patients underwent 3-6 operations with additional distraction before the main surgery.

### Pre-operative data

The mean age at the time of spine fusion was 13.8 ± 3 years (range 7-18 years).

The mean preoperative thoracic and lumbar curve were 109.5 ± 19.9° (range 83° - 142°) and 75.6 ± 26.7° (range 40° - 108°) respectively. Preoperative flexibility of scoliosis curve was smaller in thoracic than in lumbar region and equaled 22.0 ± 11.6% (range 2.5 - 42.3%) and 27.2 ± 5.2% (range 18 - 32%) respectively. The mean apical vertebral rotation was 76.8 ± 34.2 mm (range 18 - 140 mm) in thoracic spine, and 25.5 ± 15.4 mm (range 0 - 43 mm) in lumbar spine.

The mean absolute value of the distance Th1-CSL was 15 ± 12.4 mm (range 0 - 37). The mean preoperative trunk shift was 17.0 ± 19.0 mm (range -20 - 45 mm). Preoperative thoracic kyphosis and lumbar lordosis were 89.1 ± 31.2° (range 32° - 150°) and 55.9 ± 17.3° (20° - 78°) respectively. The value of the Th12-L2 angle varied significantly, with mean preoperative angle 2.6 ± 23.2° (range -36° - 40°).

### Spine fusion

We performed posterior spine fusion alone on 6 patients and combined anterior and posterior fusion (one- or two stage) on 5 cases. Additionally, at the same time thoracoplasty was carried out to improve rib hump in 2 cases. Posterior segmental spinal instrumentation was applied in all cases, with use of hooks, pedicular screws, sublaminar or translaminar wires - depending on technical possibilities and when patients were operated on. The mean operation time was 4.2 ± 2.6 hours (range 2.5 - 6 hours). Number of fused vertebra was 11.4 ± 2.6 (range 6 - 14) on average. The mean blood loss was 818 ± 520 ml (range 200 - 1800 ml). There were no intraoperative complications.

During the follow up 4 patients underwent reoperations due to increasing imbalance, instrumentation failure - primary causes were pseudoarthroses in the fused area. Costoplasty improving trunk shape was performed in 2 patients during the follow up.

### Post-operative data

**Figure 1:**
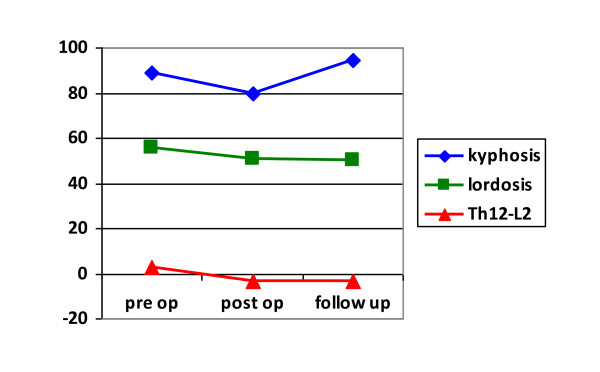
**The results of sagittal parameters: kyphosis, lordosis and Th12-L2 angle**.

The mean postoperative thoracic and lumbar curve improved to 79.3 ± 16.1° (range 56° - 105°) and 58.5 ± 27.7° (range 10° - 95°) respectively. The average thoracic and lumbar correction percentage was 26.4 ± 14.9% (range 5.3 - 50.4%) and 26.3 ± 21.2% (range 7.9 - 75%) respectively. There was slight loss of correction during the follow up period, and finally mean thoracic and lumbar curve was 85.2 ± 12.6° (range 65° - 100°) and 60.8 ± 23.7° (range 15° - 88°) at last examination. Final correction equaled 20.1 ± 16.6% (range 0 - 50.4%) and 22.2 ± 25.6% (range -15.8 - 62.5%) respectively. Other coronal plane results are presented in Table 1(Table 1). After surgery, the mean kyphosis was 79.5 ± 40.3° (range 21° -170°), and lordosis was 50.8 ± 18.6° (range 20° -79°). Thoraco-lumbar junction was slightly lordotic or plane, and mean Th12-L2 angle was -3.5 ± 9.9° (range -19° - 15°). Hyperkyphosis increased during the follow up and at last examination mean kyphosis was 94.5 ± 43.6° (range 18° -180°) (additional file 1). Lordosis remained stable during the follow up and was 50.2 ± 28.2° (range 12°-90°) on average. The mean Th12-L2 angle did not change significantly during the follow up and at last examination it was -3.1 ± 22.2° (range -40° - 40°) (Figure 1).

**Table 1:** Coronal balance data before and after treatment

	Before surgery	After surgery	After follow up
Thoracic AVT	76.8 ± 32.5 mm (range 18-140 mm)	62.5 ± 29.3 mm (range 21-120 mm)	59.4 ± 17.2 mm (range 35- 82 mm)

Lumbar AVT	25.5 ± 15.4 mm (range 0-43 mm)	30.4 ± 18.7 mm (range 3- 63 mm)	33.9 ± 21.7 mm (range 0- 64 mm)

Absolute Th1-CSL value	15 ± 13.1 mm (range 0-37 mm)	32 ± 18.4 mm (range 5-60 mm)	27 ± 21.8 mm (range 3-60 mm)

Trunk shift	17.0 ± 20.5 mm (range -20-45 mm)	8.8 ± 18.3 mm (range -20 - 30 mm)	19.3 ± 15.2 mm (range 0 - 45 mm)

AVT = apical vertebral rotation

## Discussion

**Figure 2:**
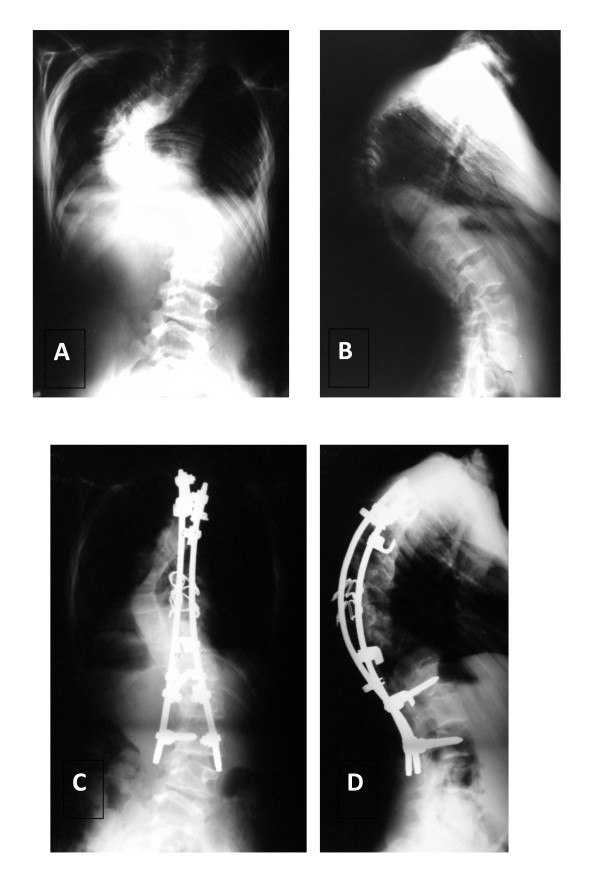
**Patient No1, boy, age at surgery: 14 yrs**. Good result after 4 years of follow up. A- AP radiogram before surgery: Cobb angle 105°, B- L radiogram before surgery: kyphosis angle 116°, C- AP radiogram 4 years after surgery: Cobb angle 60°, D- L radiogram 4 years after surgery: kyphosis 82°.

**Figure 3:**
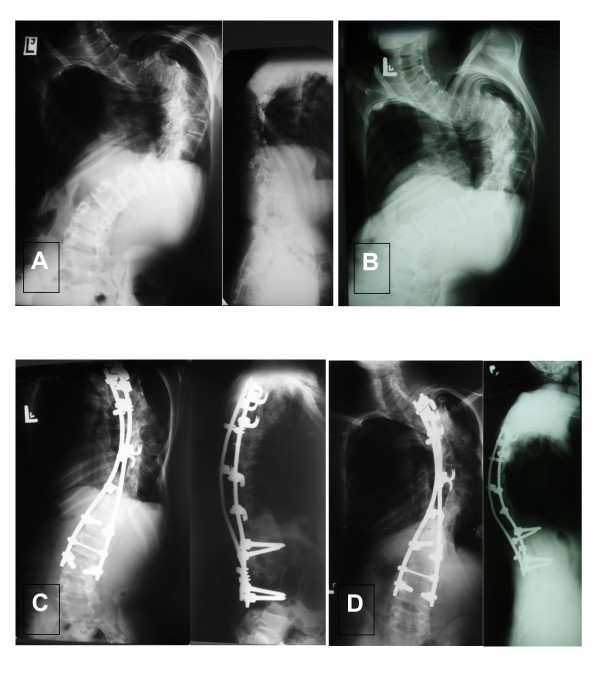
**Patient No11, male, age at surgery: 17 yrs**. A - AP and L radiograms before surgery, scoliosis angle: 135°, B- radiogram - an oblique view (acc. to Stagnara), C- AP and L radiograms after surgery, D- AP and L radiograms 6 years after surgery. Quite good result, although both coronal and sagittal balance is not ideal.

**Figure 4:**
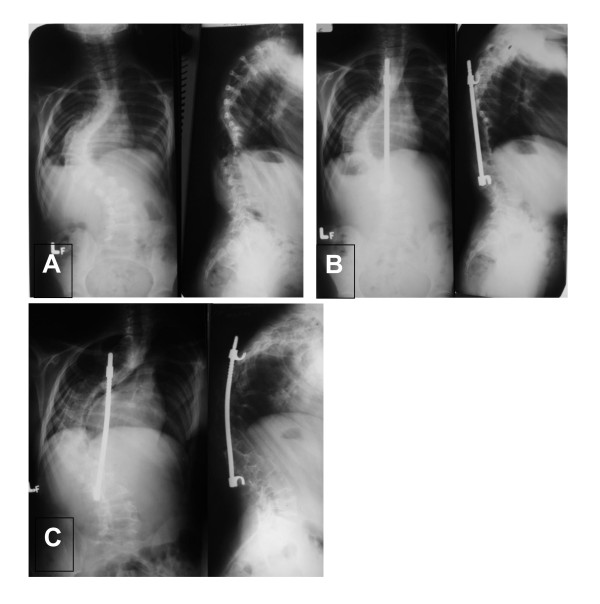
**Patient No7, female, age at spinal fusion: 12 yrs**. A- first AP and L radiograms at the age of 5; B - correction without fusion (Moe technique); C- at the age of 12, just before spinal fusion. Hyperkyphosis above instrumentation.

**Figure 5:**
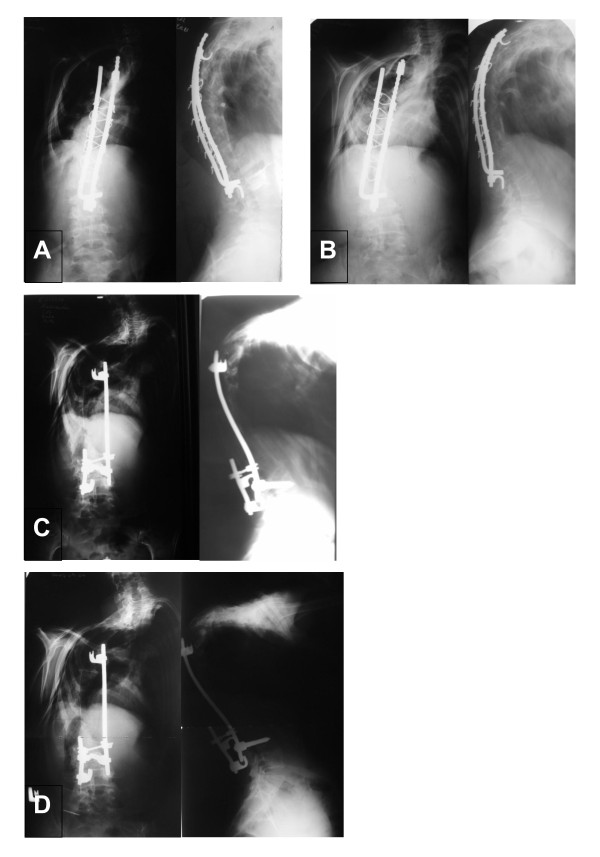
**Patient No7, next radiograms:A** - after spinal fusion with posterior instrumentation (still kyphosis in upper thoracic region - too short extent of fusion?); **B** - at the age of 14, spinal decompensation; C- after reoperation: costoplasty, removal of instrumentation, new posterior stabilization at the area of pseudoarthrosis; D- last examination - at the age of 21. Hyperkyphosis, sagittal imbalance.

Etiopathogenesis of collagen defects in Ehlers Danlos syndrome has been already described quite well. One of the most striking symptoms of the disease is kyphoscoliosis, seen in type IV of syndrome. In our patients the onset of spine deformity was assessed below 4^th ^year of life, similarly to patients of McMaster [4]. This deformity is caused by muscle hypotonia and loose ligaments and it can increase to a huge degree. To our knowledge conservative treatment is ineffective. Operative treatment of kyphoscoliosis in Ehlers Danlos syndrome is difficult, and described by other authors "satisfying result" meant certain correction with deformity stabilization, without perfect restoration of sagittal contour [4,7]. Main preoperative curve exceeded 80° in all our patients. Thus, curves were severe with additional disturbances of sagittal contour (Figure 2, 3, 4, 5). Achieved correction of scoliotic deformity was not big, only just above 26%, comparable to spinal elasticity. Bigger correction was reported by McMaster and Yang, but their patients had minor degree of deformity [4,8]. Also brilliant correction was described by Russian authors on 8 patients, but detailed information about this patients' group was not available for us [9]. Blood loss did not exceed 2000 ml in our group and no vascular complications were observed - neither avulsion of segmental arteries via anterior approach nor tear of external iliac artery [5-8]. We did not see any temporary or permanent neurological complications. Coronal plane correction remained stable during follow up and the smaller preoperative curve, the bigger percentage of correction was obtained. Sagittal balance remained a challenging problem; starting with first radiograms, the significant tendency for hyperkyphosis was observed. We found that all 5 patients treated primarily with correction without fusion had increasing kyphosis above the upper end of instrumentation. Generally, kyphosis was stiff, with poor correction during spondylodesis. During follow up sagittal profile worsened, although lordosis and Th12-L2 did not changed significantly, we observed increasing hyperkyphosis. Further reoperations were bound with sagittal imbalance. It is worth emphasizing that due to low frequency of Ehlers-Danlos syndrome, this retrospective study covers years between 1990 and 2007, thus possible operative techniques were limited in the beginning and varied during time.

## Conclusions

Huge spinal deformities in patients with Ehlers-Danlos syndrome require complex and extensive surgery.

There is a big risk of sagittal imbalance in this group.

## Competing interests

The authors declare that they have no competing interests.

## Authors' contributions

BJ conceived of the study, participated in collecting radiologic data and drafted the manuscript

TP participated in the design of the study and performed the statistical analysis in cooperation with KL

MT conceived of the study and participated in its design and coordination

KL participated in collecting radiological data and participated in statistical analysis

All authors read and approved the final manuscript.

## Supplementary Material

Additional File 1 **Main data of all patients**. Microsoft Excel table with data of all patients. Click here for file
